# Clinicoradiological and Histopathological Correlation of Placenta Accreta Spectrum in a Tertiary Care Center

**DOI:** 10.7759/cureus.109024

**Published:** 2026-05-17

**Authors:** Vasuki D., Leena Dennis Joseph, Venkata Sai, Rajeswari K.S.

**Affiliations:** 1 Pathology, Sri Ramachandra Institute of Higher Education and Research, Chennai, IND; 2 Radiology, Sri Ramachandra Institute of Higher Education and Research, chennai, IND; 3 Obstetrics and Gynaecology, Sri Ramachandra Institute of Higher Education and Research, chennai, IND

**Keywords:** diagnostic imaging, histological techniques, magnetic resonance imaging, morbidity, placenta accreta spectrum

## Abstract

Background: Placenta accreta spectrum (PAS) is an obstetric condition linked to increased maternal morbidity and death. Early detection through clinical assessment and radiological imaging, particularly MRI, may be useful for prenatal diagnosis and surgical planning of selected cases of PAS. However, histological testing is still the gold standard for a conclusive diagnosis. The purpose of this study is to assess the diagnostic concordance of clinical and radiological findings with histology in PAS.

Methods: This case series was conducted in a tertiary care center and included 11 cases of histopathologically confirmed PAS. MRI finding, where available, were compared with histopathological analysis of hysterectomy specimens. Descriptive statistics were used to summarize demographics, comorbidities, and diagnostic methods.

Results: The average age of the participants was 31.9 years. The prevalent comorbidities were hypothyroidism seen in four (37%) cases, anemia seen in three (27%) cases, and gestational diabetes mellitus (GDM) seen in two (18%) cases. Clinical suspicion of PAS was present in seven of 11 (63.3%) cases, while MRI was performed in five cases and detected PAS in all five (100%) cases. Histopathology confirmed the diagnosis in all cases where clinical suspicion and MRI suggested the diagnosis.

Conclusion: In this case series, though clinical evaluation and MRI contributed to antenatal detection of PAS, definitive diagnosis relied on histopathological examination. These findings emphasize the need of a comprehensive diagnostic approach for accurately identifying and managing the PAS.

## Introduction

Placenta accreta spectrum (PAS) refers to a spectrum of disorders affecting the placentation process due to the presence of decidual dysfunction and invasive trophoblasts within the myometrium [1]. PAS occurs in various degrees, from placenta accreta, in which there is attachment of the villi to the myometrium, to placenta increta, where there is deeper invasion, to placenta percreta, in which the placenta breaches the uterine serosa [2-4].

Postpartum hemorrhage is a major cause of maternal morbidity and mortality due to severe bleeding, often requiring blood transfusion and hysterectomy [3,5,6]. There has been an alarming increase in the incidence of postpartum hemorrhage in recent times, owing to the rising rates of cesarean births and increase in maternal age [7,8].

Cesarean deliveries previously undertaken, placenta previa, multiparity, and uterine surgery have been identified as predisposing factors for PAS, and the risk increases with each previous cesarean birth [9,10].

Histopathologically, PAS is characterized by a defect in the development of the decidua basalis and Nitabuch’s layer, which results in the direct attachment of chorionic villi to the myometrium [7]. It is crucial to have standardized histopathologic classification and reporting to ensure proper diagnosis and prognosis [11].

Antenatal diagnosis of the disease is very important for optimizing maternal outcomes. Ultrasound and MRI are imaging techniques used in the diagnosis of PAS and evaluation of the degree of placental invasion [12]. MRI is especially helpful in complicated cases where placental invasion can be mapped using this technique [13,14]. Nevertheless, histopathological examination is still considered the gold standard in making a definitive diagnosis of the condition [11,15].

Correlation between clinical suspicion, radiological findings, and histopathology are crucial for improving diagnostic accuracy and optimizing management strategies. Discrepancies between imaging findings and histopathological confirmation have been reported, highlighting the need for further evaluation [16,17].

Therefore, this study aims to analyze and correlate clinical, radiological, and histopathological findings in cases of PAS managed with hysterectomy in a tertiary care setting.

## Materials and methods

This is a retrospective observational study, conducted in the Department of Pathology at a tertiary care hospital in Chennai. The study period was from January 2024 to December 2025. All histopathologically confirmed cases of PAS that were managed with hysterectomy during the study period were included in the analysis. Cases managed conservatively were excluded, as histopathological confirmation was not available for these patients. A total of 11 cases were included in the study. The study included hysterectomy specimens of cases with histopathologically confirmed PAS, in which hysterectomy was performed based on clinical and/or radiological suspicion, with the aid of available histopathological slides and complete medical records. Poorly preserved, inadequate histopathological specimens and cases without confirmatory histopathology were excluded from the study.

**Figure 1:**
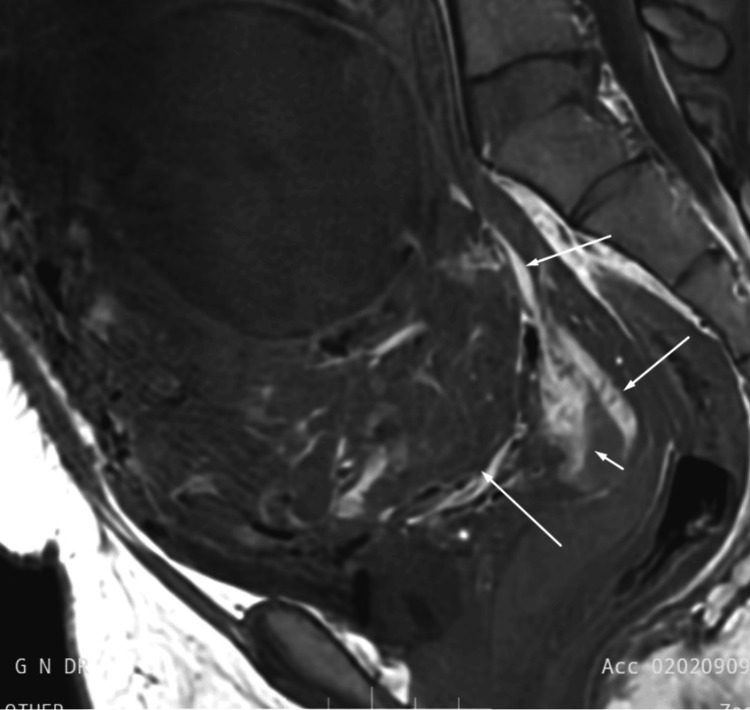
Sagittal T2-weighted MRI image showing loss of retroplacental clear space with severe thinning of myometrium (arrows) in a case of placenta previa This image corresponds to case number 6 in Table 1.

Data were retrieved from the laboratory information system (LIS) and medical records. Data pertaining to demography ,obstetric history, clinical diagnosis, radiological findings (available in five of 11 cases: ultrasound in one case and MRI in four cases, based on original clinical radiology reports without retrospective image re-evaluation), type of specimen received and histopathological findings were recorded is shown in Table 1. Representative radiological findings suggestive of PAS are shown in Figure 1.

**Table 1:** Individual clinicoradiological and histopathological characteristics of patients with PAS USG: Ultrasonography; PAS: Placenta accreta spectrum; GDM: Gestational diabetes mellitus; MTP: Medical termination of pregnancy; LSCS: Lower segment cesarean section; RPC: Retained products of conception; DM: Diabetes mellitus

S. No.	Age	Previous mode of delivery	Comorbidities	Clinical diagnosis	Radiological findings (USG/MRI findings)	Specimen sent	Histopathological diagnosis
1	34	LSCS	Nil	PAS with placenta previa grade IV	MRI findings - Bulky uterus with T2 heterogenous residual placental tissue with few focal nodular enhancement noted and partial involution	Hysterectomy specimen with placenta in situ	Placenta accreta
2	30	LSCS	Nil	Placenta previa	Not done	Hysterectomy specimen with placenta in situ	Placenta accreta
3	34	LSCS	GDM, Hypothyroidism	Placenta increta	USG findings - Loss of retroplacental clear space with severe thinning of myometrium over the fundal region	Hysterectomy specimen (uterus with cervix and adnexa)	Placenta accreta
4	28	LSCS	GDM, Hypothyroidism	Grade A placenta previa	Not done	Hysterectomy specimen (uterus with cervix and adnexa)	Placenta increta
5	37	LSCS	Ovulation induction conception, Hypothyroid		Not done	Hysterectomy specimen with placenta in situ (uterus with cervix and adnexa)	Placenta accreta
6	28	LSCS	Bleeding per vaginum, Anemia	Grade IV placenta previa with focal placenta accreta	MRI findings - Multiple intraplacental T2 hypointese bands, few focal areas of buldge in uterine contour along anteroinferior aspect of placenta, features possibly of PAS disoder	Hysterectomy specimen with placenta in situ	Placenta accreta
7	34	LSCS	Failed MTP with cesarean scar pregnancy	Scar pregnancy	Not done	Hysterectomy specimen with placenta in situ (uterus with cervix and adnexa)	Placenta accreta
8	32	LSCS	Umbilical hernia	Emergency LSCS	MRI-central placenta previa, subamniotic hemorrage in the fundal region and thin rim RPC also noted in the posteroinferior aspect. f/f/o placenta accreta	Hysterectomy specimen with placenta in situ (uterus with cervix and adnexa)	Placenta accreta with dystrophic calcification (10%)
9	41	LSCS	DM, Hypothyroid, Severe Anemia	PAS with acute postpartum hemorrhage	MRI features are in favor of PAS disoder	Hysterectomy specimen with placenta in situ (uterus with cervix and adnexa)	Placenta increta
10	28	LSCS	Polyhydraminos	PAS	Not done	Hysterectomy specimen with placenta in situ (uterus with cervix and adnexa)	Placenta percreta
11	25	LSCS	Anemia	Placenta previa	Not done	Hysterectomy specimen with placenta in situ	Placenta increta

**Figure 2:**
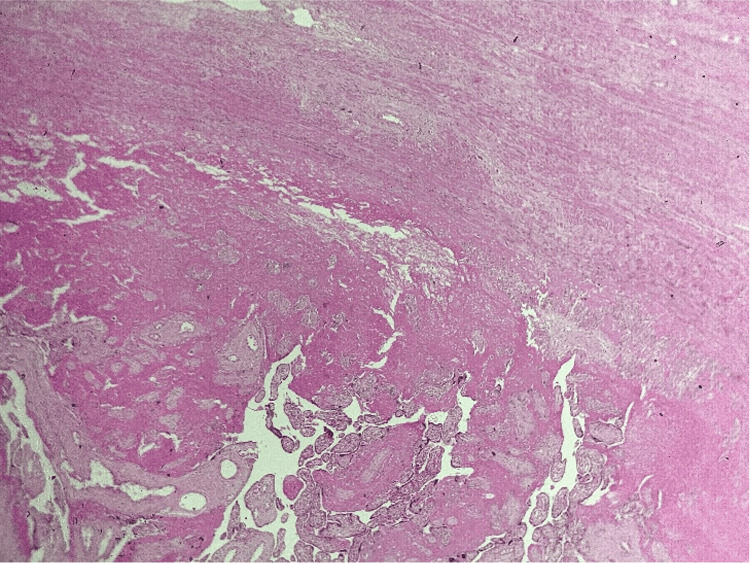
Histopathological image showing chorionic villi implantation directly on the surface of myometrium without intervening decidua suggesting placenta accreta Hematoxylin and eosin stain, ×200

**Figure 3:**
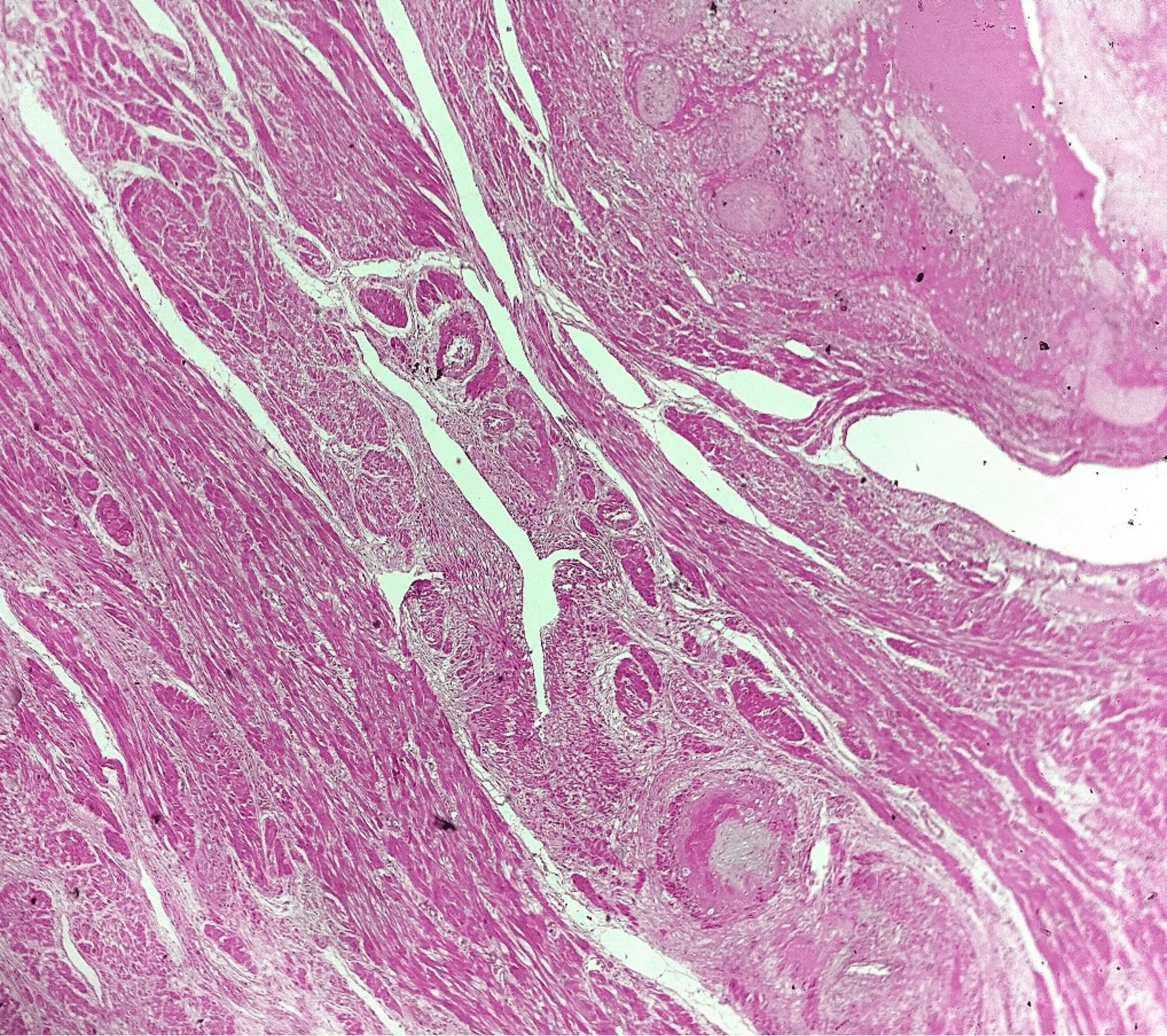
Histopathological image showing chorionic villi within the myometrium. Fibrin and extra villous trophoblast are seen present between the villi and myometrial fibres suggesting placenta increta. Hematoxylin and eosin stain, ×200

**Figure 4:**
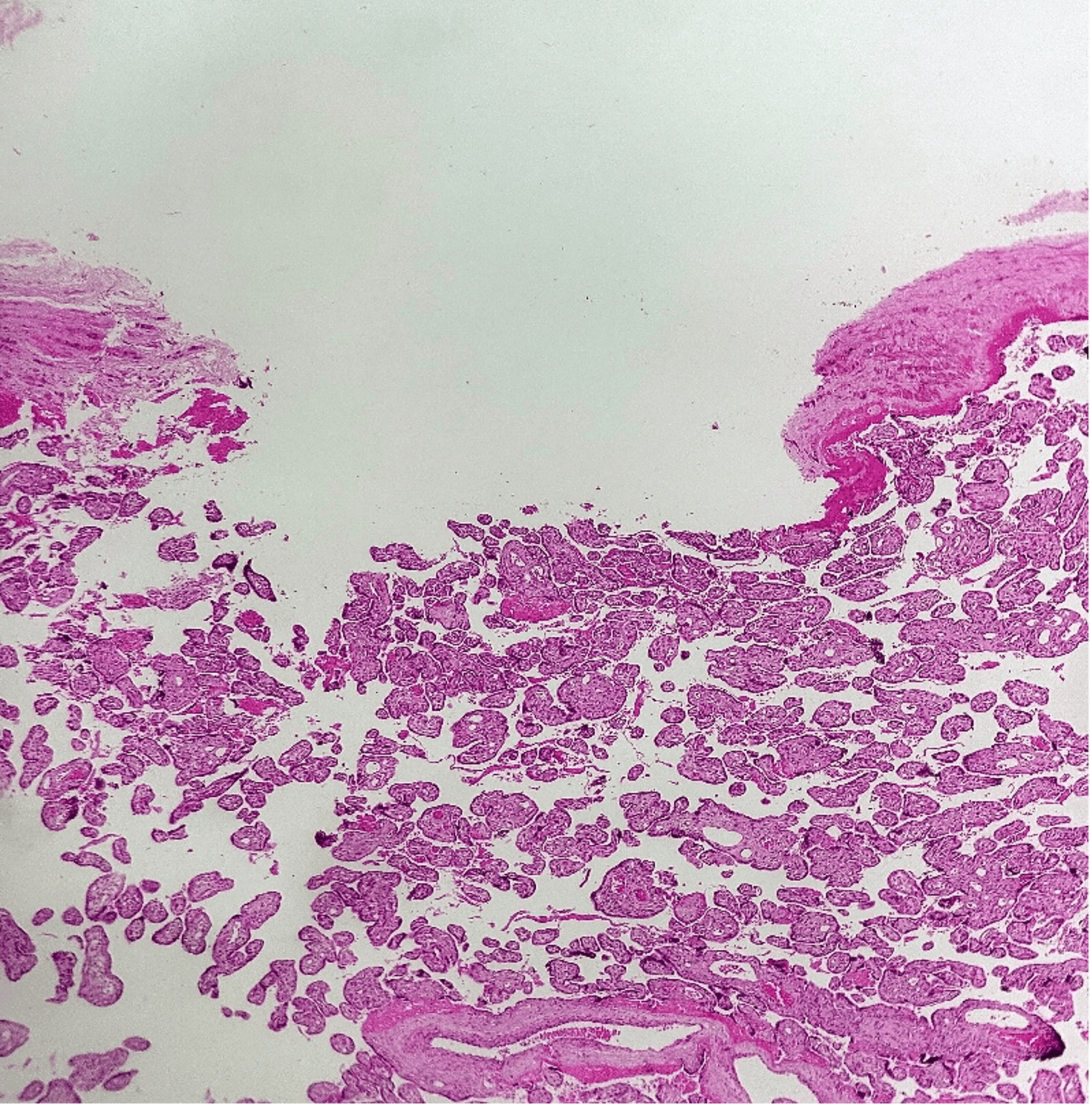
Histopathological image showing chorionic villi penetrating completely through the thinned out myometrium Hematoxylin and eosin stain, ×200

Archived paraffin samples of hematoxylin and eosin-stained glass slides prepared from formalin-fixed paraffin-embedded (FFPE) tissue blocks of diagnosed PAS in the Department of Pathology were used for histopathological examination, to classify PAS into accreta, increta, and percreta based on depth of invasion as shown in Figures 2, 3, 4, respectively. Radiological diagnosis of PAS was based on ultrasonographic and/or MRI findings including placental lacunae, loss of retroplacental clear zone, myometrial thinning, abnormal uterovesical interface vascularity, bridging vessels, uterine bulging, and evidence of placental invasion into adjacent structures.

Data were entered in Microsoft Excel and analyzed descriptively .Clinical, radiological, and histopathological findings were compared,concordance was assessed as agreement between radiological suspicion of PAS and histopathological confirmation. Results were expressed as frequencies, percentages, and mean values.

The Institutional Ethics Committee approval was obtained (CSP-MED/26/MAR/126/83). Waiver of informed consent was granted due to the retrospective study design. Patient confidentiality and anonymity were strictly maintained.

## Results

**Figure 5:**
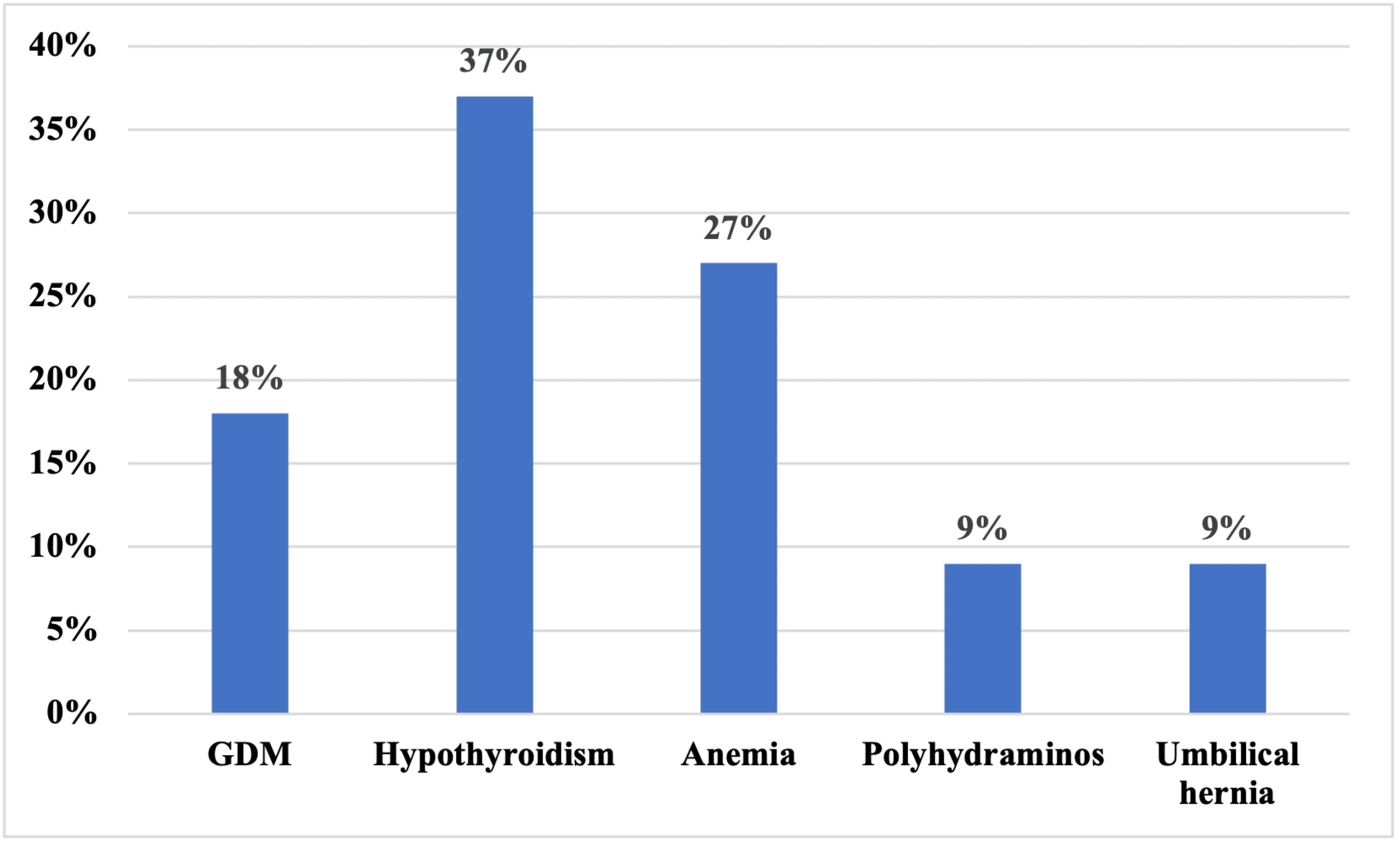
Distribution of comorbidities recorded as descriptive clinical features in patients with PAS (n = 11) GDM: Gestational diabetes mellitus; PAS: Placenta accreta spectrum

A total of 11 cases of PAS confirmed by histopathological examination were included in this case series (Table 1). The mean age of the study participants was 31.9 years. The distribution of comorbidities among the participants is shown in (Figure 5). The most common comorbidity was hypothyroidism in four (37%) patients, followed by anemia in three (27%) cases and gestational diabetes mellitus (GDM) in two (18%) cases. All 11 (100%) cases had a history of previous lower segment cesarean section (LSCS). Less frequent conditions included polyhydramnios and umbilical hernia, each accounting for one (9%) case. The comorbidities observed in the study cohort are presented descriptively and were not interpreted as risk factors for PAS. Out of the 11 histopathologically confirmed cases, seven (63.6%) cases were clinically suspected, while five (45.5%) cases were diagnosed using radiological imaging, including one case diagnosed on ultrasound and four cases on MRI as shown in Figure 1 (Table 2). Clinical assessment and radiological imaging contributed to preoperative suspicion of PAS; however, not all histopathologically confirmed cases were identified by either modality. This suggests that both clinical and radiological methods have limited sensitivity when used independently.

**Table 2:** Mode of diagnosis

Mode of diagnosis	Frequency
Number of cases with clinical suspicion	7
Number of cases radiologically diagnosed	5
Histopathologically confirmed	11

## Discussion

PAS is now emerging as one of the significant causes of morbidity in obstetrics because of its tendency to cause massive bleeding and surgical problems. All included cases were histopathologically confirmed PAS, while clinical suspicion and radiological detection were incomplete and variably documented.

In most cases, it was observed that the history of previous cesarean delivery played an important role, which is well documented in the literature as being the most important risk factor for developing PAS [7,9]. The same findings were also obtained by Thurn et al., who showed a very strong correlation between multiple cesarian deliveries and PAS [18]. It further proves the hypothesis that uterine scars have a predisposition to develop abnormal trophoblast implantation. Unlike other studies that have reported higher parity as a risk factor for PAS [8], our study was not designed to evaluate risk factors, and no conclusions regarding such associations can be drawn due to the small sample size.

Radiological evaluation, predominantly MRI where available, supported clinical suspicion in five cases; however, imaging findings were variably documented and not available for all patients, with histopathology remaining the definitive diagnostic standard. This is consistent with the results shown by D’Antonio et al. wherein the sensitivity of prenatal imaging in detecting invasive placentation was noted to be high [13]. Likewise, according to Collins et al., imaging plays a significant role in preoperative planning and risk assessment [16]. In this study, there were some cases wherein MRI results were inconsistent with histopathologic analysis, implying either an overdiagnosis or underestimated extent of invasion. Such inconsistencies have also been previously documented by authors like Jauniaux et al. [17].

Pathologically, majority of patients in our study were found to be within the PAS in relation to the extent of invasion into the myometrium. This classification system conforms to guidelines provided by Hecht et al., which recommend standardization in reporting to ensure proper diagnosis and prognosis [11]. The pathological basis of PAS identified in this study, such as the absence of decidua basalis and atypical attachment of villi, conforms to the pathophysiology described by Jauniaux et al. [7]. Similar histopathological findings were described in the study by Dutta et al., where "trophoblastic tissue invading the myometrium without intervening decidua formed the pathological basis of PAS" [19].

Clinically, several cases were suspected preoperatively, but histopathology remained the definitive method for confirming PAS. This implies that the diagnostic precision in clinical assessment was moderate. Similar trends have been noted in other studies, where reliance on risk factors alone contributed to overdiagnosis [20].

However, there were also instances where the cases did not have strong clinical suspicion, but they were later proven histologically. While there were some cases with concurrence among the diagnostic modalities, variations have been observed when it comes to the grading of invasive depths, that is, accreta, increta, and percreta. These results are consistent with the findings made by Stanzione et al., wherein they found out that imaging was less reliable especially in terms of discriminating deeper invasions due to certain factors [21].

In conclusion, the results obtained from this study are in harmony with current knowledge regarding the need for multimodal evaluation in managing PAS.

Limitations

A limitation of this study is the incomplete availability of clinical details, such as presenting symptoms and parity, due to its retrospective design. In addition, the small sample size (n = 11) limits the generalizability of the findings. This is especially true because a retrospective study is inevitably prone to incomplete or missing data, compromising the accuracy of the results. Only hysterectomy specimens with histopathologically confirmed PAS were included, introducing selection bias toward more severe cases and preventing evaluation of false-positive clinical or radiological diagnoses. Variability in imaging modality (MRI or ultrasound), lack of interobserver assessment of imaging findings, and the fact that MRI was not performed in all cases, limiting conclusions regarding MRI detection rates or diagnostic performance also pose potential limitations to the study. Maternal outcome information such as blood loss, admission to ICU, and maternal morbidity was also absent, limiting the practicality of the study. Lastly, the sample size was too small for determining the predictive value of imaging in placenta accreta, as indicated by sensitivity and specificity.

## Conclusions

Due to an increase in cesarean section rates, PAS continues to be a major obstetric problem. Clinical and radiological findings showed agreement with histopathological diagnosis in a subset of cases; however, no formal statistical measure of agreement (such as Cohen’s kappa) was performed, and histopathology remained the diagnostic gold standard.

There are still differences in determining the level of invasion, despite the fact that imaging modalities like MRI are useful for prenatal diagnosis and surgical planning. Although clinical suspicion based on risk factors may lead to both over- and under-recognition of PAS, this study included only histopathologically confirmed cases; hence, diagnostic overestimation or underestimation could not be evaluated.

Our results highlight the significance of a multidisciplinary approach that incorporates radiographic, histological, and clinical examination for precise PAS diagnosis and treatment. To increase diagnostic precision and standardize procedures, more extensive prospective research is needed.
